# Integration of Microstrip Slot Array Antenna with Dye-Sensitized Solar Cells

**DOI:** 10.3390/s20216257

**Published:** 2020-11-02

**Authors:** Bowen Bai, Zheng Zhang, Xiaoping Li, Chao Sun, Yanming Liu

**Affiliations:** The Key Laboratory of Information and Structure Efficiency in Extreme Environment, The Ministry of Education of China, and The School of Aerospace Science and Technology, Xidian University, Xi’an 710071, China; zzhang_1992@stu.xidian.edu.cn (Z.Z.); xpli@xidian.edu.cn (X.L.); sunc@xidian.edu.cn (C.S.); ymliu@xidian.edu.cn (Y.L.)

**Keywords:** dye-sensitized solar cells, integration, antenna array, solar antenna

## Abstract

This paper describes the integration of microstrip slot array antennas with dye-sensitized solar cells that can power array antennas at 5.8 GHz, ensuring normal communications. To appraise the antennas, a 2 × 2 circularly polarized microstrip slot array antenna integrated with dye-sensitized solar cells using a stacked design method was analyzed, fabricated and measured. The size of the entire array is 140 mm × 140 mm, where the size of each solar cell is 35 mm × 35 mm. The results show that the effect of the antenna has a slight influence on the output performance of the solar cells, and the interference of the output current of the solar cells to the antenna feeding system is negligible. The gain of the array antenna increases by 0.12 dB and the axial ratio is reduced to 1.50 dB after the integration of dye-sensitized solar cells. The integration saves a lot of space, and has the ability of self-sustaining power generation, thus providing reliable and long-term communication for various communication systems.

## 1. Introduction

Recently, solar energy has received more and more attention as a clean renewable energy source, and the solar antenna (solant) has drawn a large amount of concern because it can not only transmit and receive electromagnetic waves, but also generate electricity [1]. The research on the fusion technology of solar cells and antennas can be traced back to 1995. Tanaka et al. [2] took the lead in designing a fusion device of solar cells and patch antenna, and the device was successfully applied to a microsatellite. Compared with the simple juxtaposition of the antenna and solar cell, the integration of the antenna and the solar cell has certain advantages in volume, weight, appearance and electrical performance. Both amorphous (a-Si) and crystalline (c-Si)-type silicon solar cells with integrated antennas have been reported [3,4,5,6,7,8]. A single crystal silicon solant is proposed [9]; several solar cells are placed 5 mm above the microstrip slot antenna, indicating a poor combination between the solar cells and the antenna. S. V. Shynu et al. [10] integrated a double-slot antenna with an amorphous silicon solar cell by covering a dual-band WLAN. K Yang et al. [11] replaced the copper grounding plane of the vivaldi antenna with an amorphous silicon solar cell; while the solant achieves a high degree of integration, there is a certain interference between the solar cell and the antenna. M. Danesh et al. [12] used a monopole antenna in combination with an amorphous silicon solar cell, and placed only the solar cell in the radiating portion of the monopole antenna, resulting in low space utilization. Therefore, it is still a challenge to integrate antennas with the solar cell to the greatest extent and eliminate the interference between antennas and solar cells.

Dye-sensitized solar cells have been widely studied and applied [13,14,15] due to their lower processing cost compared with crystalline silicon cells. With the development of research, the conversion efficiency of dye-sensitized solar cells has been improved [16], and can be printed on flexible conductive plastic layers for enhanced integration [17,18]. However, there is little literature on the integration of antennas with dye-sensitised solar cells. The first dye-sensitised solar cell antenna in a proof-of-concept dipole configuration was studied in [19]; series-connected dye-sensitised cells could produce 1.49 V and 15.5 mA, which meets design requirements. However, the interference caused by the integration of antennas with dye-sensitised solar cells still needs to be analyzed. A compatible integration of a circularly polarized omnidirectional metasurface antenna with solar cells has been reported in [20]. While the antenna gain of type IV is 4.1 dBi, it can be predicted that the output power generated by the solar cell array is not high due to the fact that the solar cells are not connected to each other, which limits its practical application.

In this paper, an integration of a 2 × 2 circularly polarized microstrip slot array antenna with dye-sensitized solar cells is designed. A novel stack design method makes the solar cell and array antenna well integrated and the simulation and measurement results show that the gain of the array antenna increased by 0.12 dB, reaching 6.60 dBi, and the axial ratio was reduced to 1.50 dB after the integration of dye-sensitized solar cells. The solar cells and the microstrip slot array antenna are perfectly integrated. The integration saves a lot of space, especially when the proposed antenna is used in satellite communication. Compared with the existing circularly polarized microstrip slot array antenna, the proposed antenna adds the output of dye-sensitized solar cells into the voltage regulation circuit to form a stable power supply for the radio frequency system, which ensures the operation of the microstrip slot array antenna. In other words, the antenna has the ability of self-sustaining power generation capabilities, so as to provide reliable and long-term communication for the communication system when the power is not easy to obtain.

## 2. Integrated Design Array Antenna and Solar Cells

**Figure 1:**
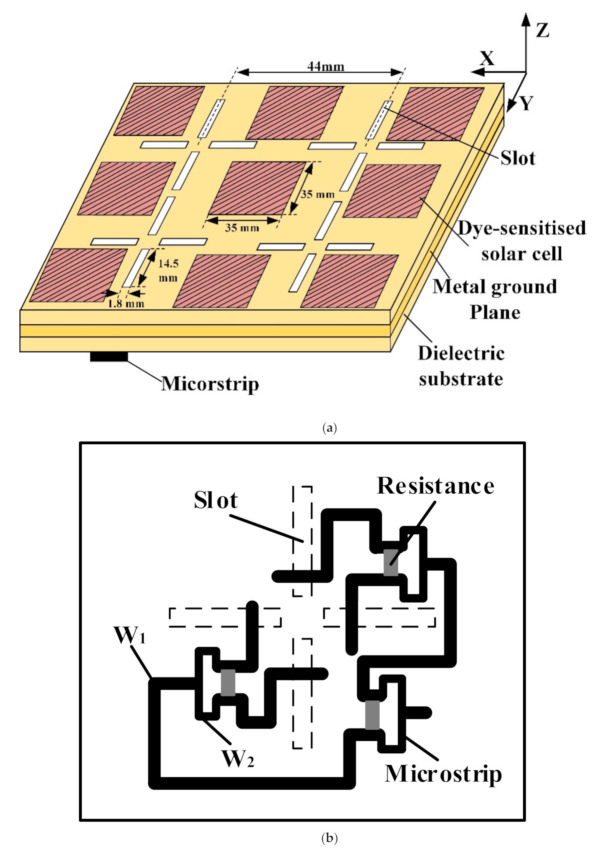
(**a**) The geometry of a 2 × 2 circularly polarized microstrip slot array antenna integrated with dye-sensitized solar cells; (**b**) microstrip feeder network.

Due to the influence of climate, environment and other factors, the linear polarization wave easily causes polarization deflection loss. These factors have little effect on the polarization deflection of circularly polarized waves, and, concerning circularly polarized antennas, polarization mismatch does not easily occur. In order to suit practical applications, a circularly polarized microstrip slot antenna is used in this paper. The geometry of a 2 × 2 circularly polarized microstrip slot array antenna integrated with dye-sensitized solar cells is illustrated in Figure 1a. The size of the entire array is 140 mm × 140 mm, where the size of each solar cell is 35 mm × 35 mm. The circularly polarized waves are excited by arranging the four slots crosswise and designing the microstrip feeder network reasonably.

The dielectric substrate of the microstrip slot antenna uses a Rogers R4350b plane, a dielectric constant of 3.48, a dielectric loss angle of 0.0037 and a thickness of 0.5 mm. The equal division Wilkinson power divider is used in the feed network to obtain the excitation signal with equal amplitude and phase difference of 90°, as shown in Figure 1b. The isolation resistance is 100 Ω, and the impedance of the feed port is set to 50 Ω. Through optimization analysis, the width of microstrip lines is W1 = 1.15 mm (50-Ω) and W2 = 0.63 mm (70.71-Ω). The slot size is 14.5 mm × 1.8 mm, and the eccentric distance of the feed point is 3 mm. The microstrip slot array antenna gain and the available solar cell area on the antenna surface need to be taken into consideration, concerning a slot spacing of 44 mm.

Dye-sensitized solar cells are a new type of photovoltaic technology developed by simulating the principle of plants in nature using solar energy for photosynthesis. DSSCs are based on dye sensitizers and nano-TiO_2_, which can make the photoelectric conversion efficiency reach a better level. At the same time, dye-sensitized solar cells (DSSCs) are rich in raw materials, non-polluting and low in cost; the manufacturing cost is only one fifth to one tenth that of silicon solar cells [21]. Therefore, dye-sensitized solar cells were chosen for this paper. Dye-sensitized solar cells adopt a stacked structure similar to the microstrip slot antenna, so the metal ground plane of the microstrip slot antenna can be used as the substrate of the solar cell.

**Figure 2:**
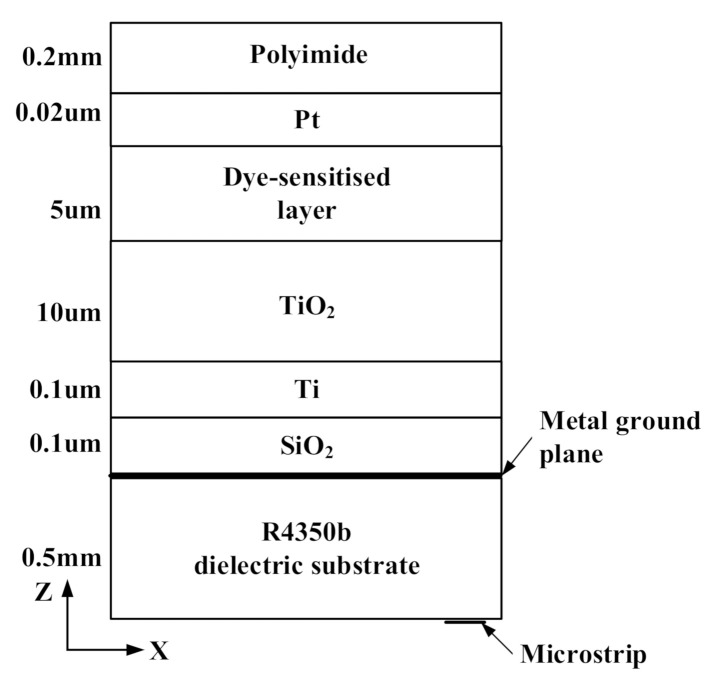
Component of the integration of a circularly polarized microstrip slot antenna with a dye-sensitized solar cell.

The integrated design does not change the intrinsic structure of the antenna, and will minimize the impact of the antenna. Due to the conductive material in the dye-sensitized solar cell, it will block the radiation of electromagnetic waves, so it is necessary to retain the slot of the antenna. The dye-sensitized solar cell and the microstrip slot antenna share a metal ground plane, and the cell structure is as described in [22]. The Components of the integration of a circularly polarized microstrip slot antenna with a dye-sensitized solar cell are shown in Figure 2. The dielectric constant and conductivity of the dye-sensitized solar cell materials at room temperature are shown in Table 1. The dye-sensitized layer is an electrolyte containing I^−^ and I^−3^, mixed with sensitizers, potassium chloride, etc. 

According to Figure 2 and Table 1, for the antenna structure shown in Figure 1, the following two spectral domain integral equations can be obtained by using the boundary condition that the total electric field tangential direction of the antenna surface is 0:

(1) $$\iint \left[{\tilde{G}}_{xx}{\tilde{J}}_{x}+{\tilde{G}}_{xy}{\tilde{J}}_{y}\right]\exp\left\{-j(k_{x}x+k_{y}y)\right\}dk_{x}dk_{y}=\iint {\tilde{G}}_{xz}{\tilde{J}}_{z}\exp\left\{-j(k_{x}x+k_{y}y)\right\}dk_{x}dk_{y}$$

(2) $$\iint \left[{\tilde{G}}_{yx}{\tilde{J}}_{x}+{\tilde{G}}_{yy}{\tilde{J}}_{y}\right]\exp\left\{-j(k_{x}x+k_{y}y)\right\}dk_{x}dk_{y}=\iint {\tilde{G}}_{yz}{\tilde{J}}_{z}\exp\left\{-j(k_{x}x+k_{y}y)\right\}dk_{x}dk_{y}$$

where ${\tilde{G}}_{xx}$, ${\tilde{G}}_{xy}$, ${\tilde{G}}_{xz}$, ${\tilde{G}}_{yx}$, ${\tilde{G}}_{yz}$, ${\tilde{G}}_{yy}$ are the components of Green’s function in the electric field spectral domain, ${\tilde{J}}_{x}$ and ${\tilde{J}}_{y}$ are the x and y spectral components of the unknown current on the surface, and ${\tilde{J}}_{z}$ are the spectral components of the feed current of the coaxial probe. The antenna is covered with multi-layer dielectric plates, where each layer of the dielectric plate is a lossy medium, their relative dielectric constant is $\varepsilon_{ri}$, the thickness of each covering layer is $h_{i}$ and the permeability of each layer of the medium is $\mu_{0}$. According to reference [23], the analytical calculation formula of spectral domain Green’s functions of the electric field on the antenna surface can be obtained as follows:

(3) $${\tilde{G}}_{xxi}(k_{x},k_{y})=k_{0}\eta_{0}[B^{h}k_{y}^{2}/k_{t}^{2}+B^{e}k_{x}^{2}/k_{t}^{2}]$$

(4) $${\tilde{G}}_{xyi}(k_{x},k_{y})=k_{0}\eta_{0}k_{x}k_{y}/k_{t}^{2}[B^{e}-B^{h}]$$

(5) $${\tilde{G}}_{yxi}(k_{x},k_{y})={\tilde{G}}_{xyi}(k_{x},k_{y})$$

(6) $${\tilde{G}}_{yyi}(k_{x},k_{y})=k_{0}\eta_{0}[B^{h}k_{x}^{2}/k_{t}^{2}+B^{e}k_{y}^{2}/k_{t}^{2}]$$

(7) $${\tilde{G}}_{xzi}(k_{x},k_{y})=jk_{0}\eta_{0}B^{e}k_{x}/\gamma^{2}$$

(8) $${\tilde{G}}_{yzi}(k_{x},k_{y})=(k_{y}/k_{x}){\tilde{G}}_{xzi}(k_{x},k_{y})$$

where:

(9) $$B^{h}=(A_{11}^{h}+A_{21}^{h})/U^{h}$$

(10) $$\left[\begin{array}{cc} \left(A_{11}^{h}\right) & \left(A_{12}^{h}\right)\\ \left(A_{21}^{h}\right) & \left(A_{22}^{h}\right) \end{array}\right]=\prod_{i=1}^{N}\frac{1}{1+R_{i}^{h}}\left[\begin{array}{cc} e^{j\gamma_{i}h_{i}} & R_{i}^{h}e^{j\gamma_{i}h_{i}}\\ R_{i}^{h}e^{-j\gamma_{i}h_{i}} & e^{-j\gamma_{i}h_{i}} \end{array}\right]$$

(11) $$R_{i}^{h}=(\gamma_{i}-\gamma_{i-1})/(\gamma_{i}+\gamma_{i-1})$$

(12) $$R_{i}^{e}=(\varepsilon_{ri}\gamma_{i+1}-\varepsilon_{ri+1}\gamma_{i})/(\varepsilon_{ri}\gamma_{i+1}+\varepsilon_{ri+1}\gamma_{i})$$

(13) $$U^{h}=\left\{j\gamma \cot y(\gamma h)\left[\left(A_{11}^{h})+(A_{21}^{h}\right)\right]-\gamma_{1}[(A_{11}^{h})-(A_{21}^{h})]\right\}$$

(14) $$U^{e}=\left\{j(k_{0}^{2}\varepsilon_{r}/\gamma )cot\;y(\gamma h)\left[\left(A_{11}^{e})+(A_{21}^{e}\right)\right]-(k_{0}^{2}\varepsilon_{r}/\gamma )[(A_{11}^{e})+(A_{21}^{e})]\right\}$$

(15) $$\gamma =\sqrt{\varepsilon_{r}k_{0}^{2}-k_{t}^{2}}$$

(16) $$\gamma_{i}=\sqrt{\varepsilon_{r}k_{0}^{2}-k_{t}^{2}}$$

(17) $$k_{t}=\sqrt{k_{x}^{2}+k_{y}^{2}}$$

Replace the superscript ‘h’ in Equations (9) and (10) with ‘e’ to obtain the expressions for each component of $B^{e}$, $A_{11}^{e}$, $A_{12}^{e}$, $A_{21}^{e}$ and $A_{22}^{e}$. k_0_ is the free space wave number, and $\eta_{0}$ is the free space wave impedance. Equations (3)–(8) are the new analytical calculation formulas for the spectral domain Green’s function of the microstrip slot antenna structure covered by the multilayer dielectric. After obtaining the calculation formula of Green’s function in the spectral domain, the solution of integral Equations (1) and (2) can be discussed, as below. Assuming that the coaxial probe is fed at point (x_p_, y_p_) and there is a constant current I_0_ on the probe, the formula for calculating the spectral domain of the current on the probe can be obtained as follows:

(18) $$\tilde{J_{z}}=I_{0}\exp[j(k_{x}x_{p}+k_{y}y_{p})]$$

Let the unknown current on the antenna surface be:

(19) $$\tilde{J_{s}}(x,y)=J_{x}(x,y)\tilde{x}+J_{y}(x,y)\tilde{y}$$

$J_{x}(x,y)$ and $J_{y}(x,y)$ are expanded by a set of basis functions, and then Fourier transform is used to obtain the spectral domain expression:

(20) $$\tilde{J_{x}}(k_{x},k_{y})=\sum_{n=1}^{N_{x}}C_{xn}{\tilde{J}}_{xn}(k_{x},k_{y})$$

(21) $$\tilde{J_{y}}(k_{x},k_{y})=\sum_{n=1}^{N_{y}}C_{yn}{\tilde{J}}_{yn}(k_{x},k_{y})$$

${\tilde{J}}_{xn}(k_{x},k_{y})$ and ${\tilde{J}}_{yn}(k_{x},k_{y})$ in Equations (20) and (21) are the spectral domain expressions of the selected basis functions. By introducing Equations (18), (20) and (21) into Equations (1) and (2), the integral Equations (1) and (2) can be solved using the Galerkin method to obtain the current coefficients C_xn_ and C_yn_. Then the relevant characteristic parameters of the antenna can be further calculated.

**Figure 3:**
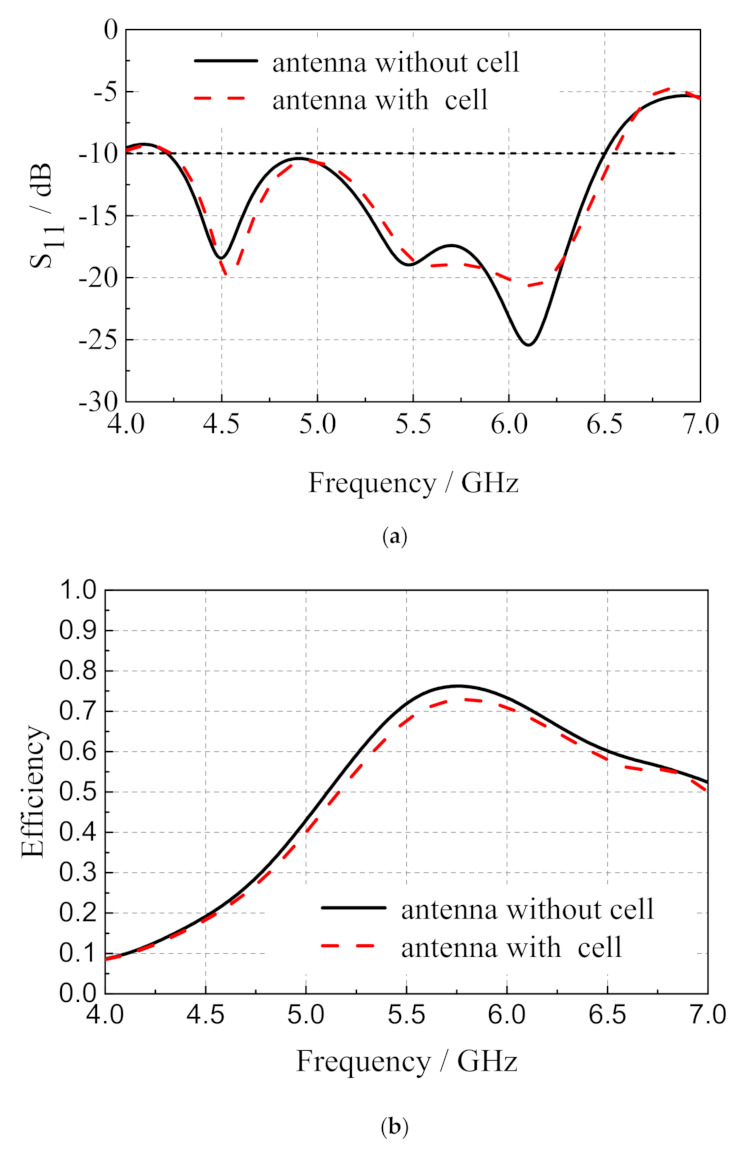
(**a**) Reflection coefficient; (**b**) radiation efficiency of the microstrip slot with or without solar cell.

The electromagnetic simulation software ANSYS HFSS is used to obtain the relevant electromagnetic parameters of the antenna [24]. The center frequency of the antenna is set to 5.8 GHz and the polarization mode is right-handed circular polarization. The microstrip slot antenna and the microstrip slot antenna integrated with dye-sensitized solar cell are simulated separately, and the related reflection coefficient and radiation efficiency are shown in Figure 3. It can be seen that the solar cell has little influence on the impedance matching of the antenna, and the microstrip slot antenna integrated with dye-sensitized solar cell has an impedance bandwidth of 2.33 GHz, from 4.23 to 6.56 GHz. After the integration of the dye-sensitized solar cell, the radiation efficiency of the antenna decreases from 76.1% to 68.9% when the operation frequency is 5.8 GHz. The radiation efficiency of the antenna decreases slightly, which indicates that the dye-sensitized solar cell has little influence on the circular polarization radiation characteristics of the antenna.

The simulation results show that the presence of the solar cell has little effect on the performance of the antenna, but the solar cell generates a corresponding current when receiving visible light irradiation. Therefore, when the cell is in working condition, the influence on the antenna performance needs to be measured.

## 3. Results and Discussion

**Figure 4:**
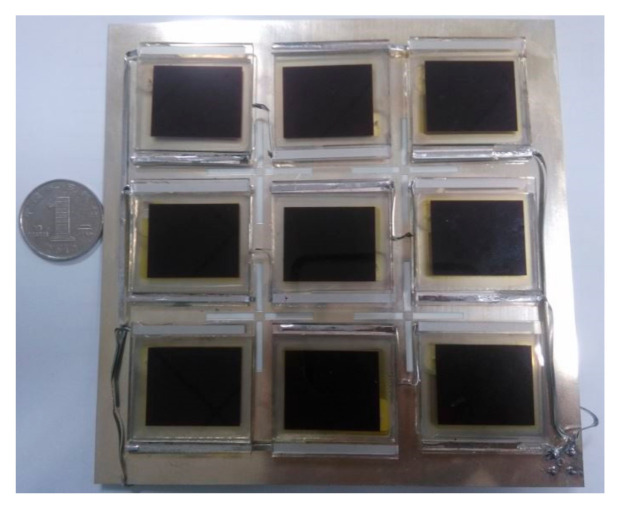
Photograph of the 2 × 2 circularly polarized microstrip slot array antenna integrated with dye-sensitized solar cells.

The photograph of the 2 × 2 circularly polarized microstrip slot array antenna integrated with the dye-sensitized solar cells is presented in Figure 4. It can be seen that the slot divides the entire antenna into nine parts, and each part is tightly connected to a dye-sensitized solar cell. Among them, three independent dye-sensitized solar cells are connected in series to improve the output voltage of the solar cells, and three series connected solar cells are connected in parallel to increase the output current of the solar cells. The dye-sensitized solar cell uses glass as the substrate, the cell and the antenna are grounded together by welding the negative electrode of the cell to the metal ground plant of the microstrip slot antenna.

**Figure 5:**
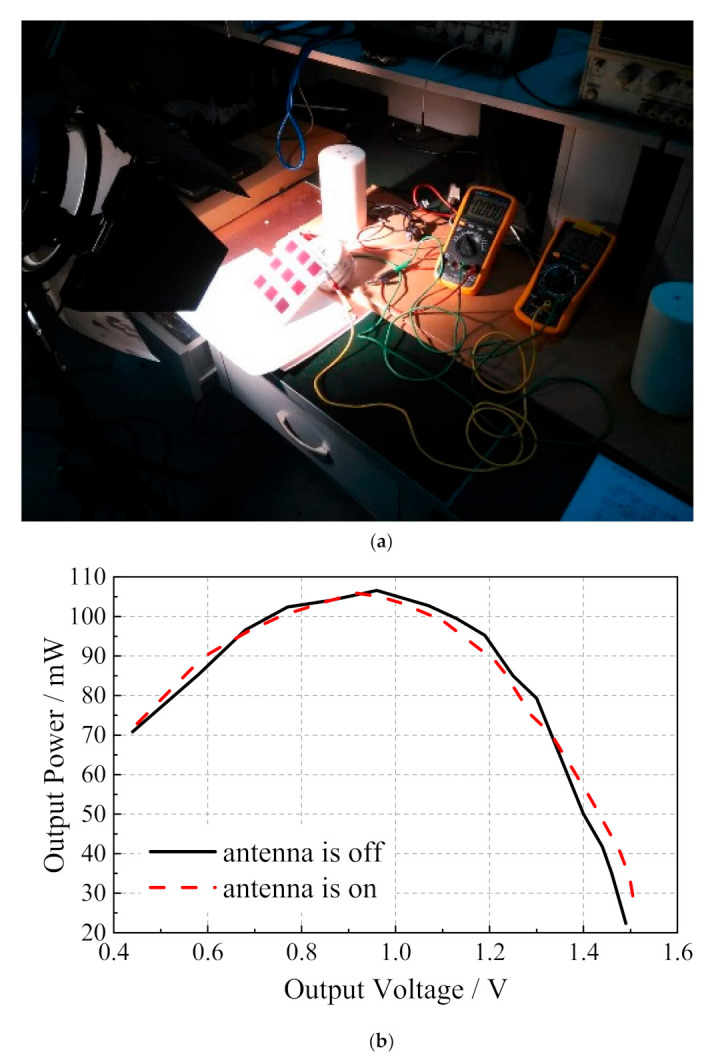
(**a**) The related experiments on the energy output characteristics; (**b**) output power versus output voltage.

**Figure 6:**
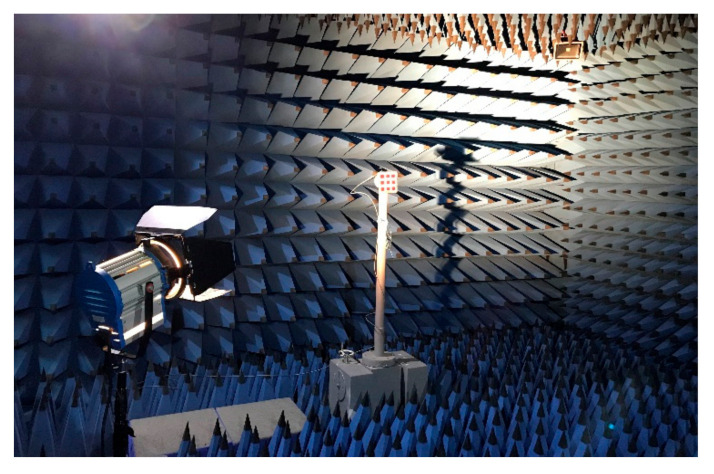
The experimental setup and the measurement environment.

The high-illuminance xenon lamp is used as the light source to illuminate the microstrip slot array antenna integrated with dye-sensitized solar cells at a suitable distance. Figure 5a shows the related experiments on the energy output characteristics of the microstrip slot array antenna integrated with the dye-sensitized solar cells. The open-circuit voltage and short circuit current of dye-sensitized solar cells are 1.94 V and 99 mA, respectively, whether the antenna works or not. The dye-sensitized solar cells are externally connected with a sliding rheostat whose resistance varies from 0 to 100 Ω. The relationship between the output voltage and the output power of the solar cell under the two conditions of antenna operation and non-operation is measured by changing the value of the sliding rheostat, as shown in Figure 5b. It can be seen that the curves coincide basically when the antenna works or does not work, which indicates that, whether it works or not, the antenna has little effect on the performance of dye-sensitized solar cells. The 2 × 2 circularly polarized microstrip slot array antenna integrated with dye-sensitized solar cells is measured in an anechoic chamber to further validate its design; the illumination intensity was maintained at 150 W/m^2^ and the relevant measurement environment is shown in Figure 6.

**Figure 7:**
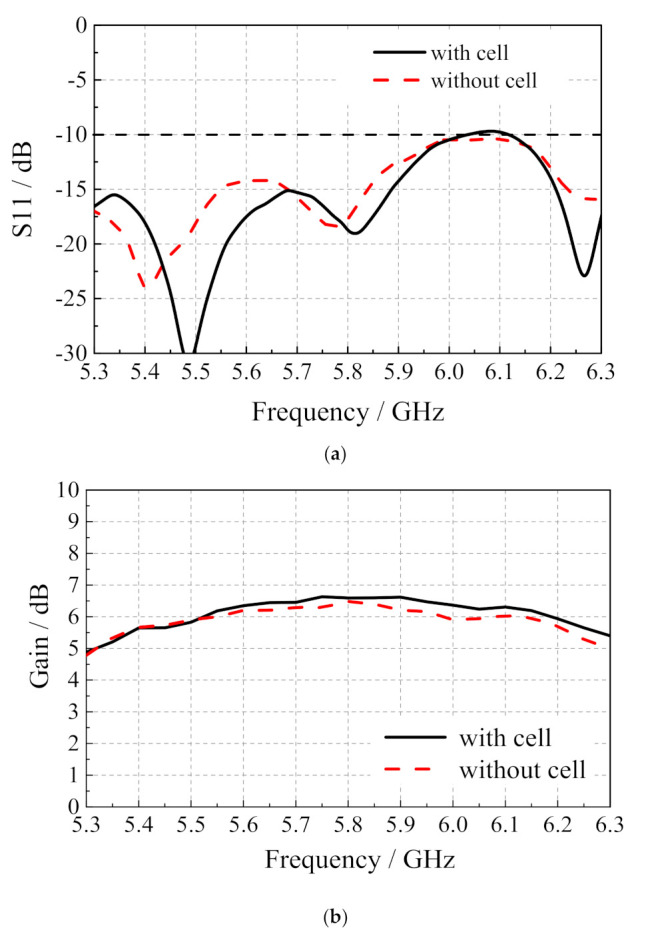
(**a**) Reflection coefficient; (**b**) gain of the microstrip slot antenna integrated with dye-sensitized solar cells varies with frequency.

Figure 7 shows the measured reflection coefficient and radiation pattern of the microstrip slot array antenna and the array antenna integrated with dye-sensitized solar cells. From the measurement results in Figure 7a, when the array antenna is integrated with dye-sensitized solar cells, the current generated when the solar cells work will have a certain influence on the reflection coefficient of the antenna, but the reflection coefficient performance of the array antenna integrated with dye-sensitized solar cells is still good on the whole. From the measurement results of Figure 7b, the gain of the microstrip slot array antenna is 6.48 dBi at 5.8 GHz, and the gain of the array antenna integrated with dye-sensitized solar cells is 0.12 dB higher than that of the microstrip slot array antenna, reaching 6.60 dBi. Generally, the existence of dye-sensitized solar cells has little effect on the gain performance of microstrip slot array antenna.

**Figure 8:**
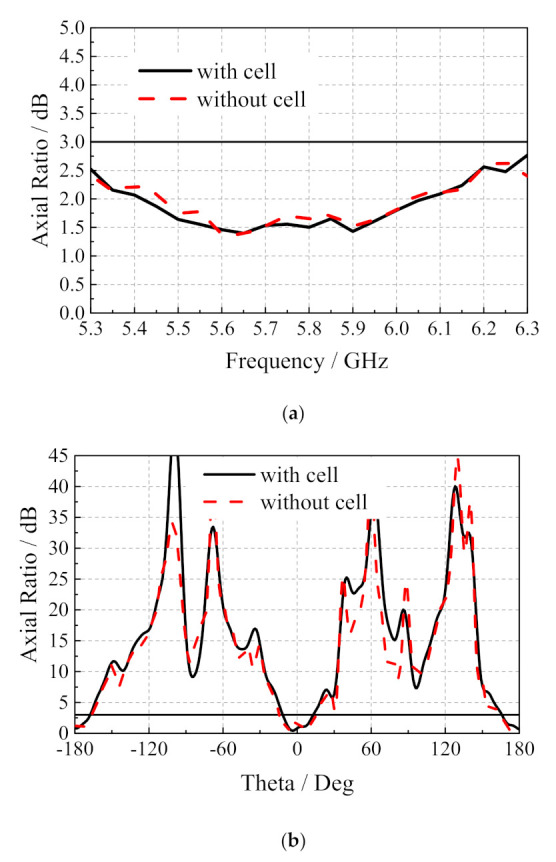
(**a**) The axial ratio varies with frequency; (**b**) axial ratio radiation pattern of the proposed antenna.

Figure 8 shows the measurement results of the influence of the axial ratio parameters of the microstrip slot array antenna integrated with dye-sensitized solar cells. It can be found from Figure 8a that the axial ratio of the microstrip slot array antenna is 1.65 dB at 5.8 GHz; the axis ratio of the array antenna integrated with dye-sensitized solar cells is 1.50 dB. The existence of dye-sensitized solar cells has little effect on the circular polarization radiation performance of the microstrip slot array antenna. The axial ratio radiation patterns in Figure 8b show that the working solar cells have almost no effect on the antenna axis ratio.

**Figure 9:**
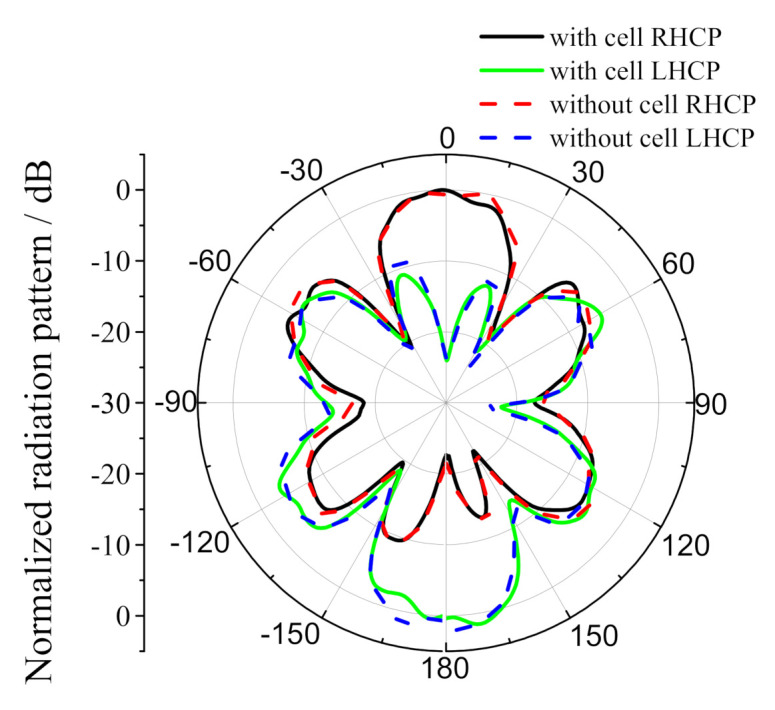
Normalized radiation patterns of the proposed antenna.

The results of normalized radiation patterns of the array antenna integrated with dye-sensitized solar cells are shown in Figure 9. It can be seen that the existence of dye-sensitized solar cells has little influence on the directivity of the microstrip slot array antenna. The above results show that, when the antenna is integrated with dye-sensitized solar cells, the measurement results in the figures will show slight changes, which are mainly caused by the interference between the solar cells and the mutual coupling between the wires. To sum up, the dye-sensitized solar cells and the microstrip slot array antenna are perfectly combined; the interference between the solar cells and the antenna is minimal. When solar cells with higher cost but better photoelectric conversion efficiency are used, the antenna size can be reduced to further improve the performance.

## 4. Conclusions

In this paper, a 2 × 2 circularly polarized microstrip slot array antenna integrated with dye-sensitized solar cells is designed. A novel stack design method makes the solar cells and the array antenna well integrated. The simulation and measurement results show that the gain of the array antenna increases by 0.12 dB and the axial ratio decreases to 1.50 dB after the integration of the dye-sensitized solar cells. Whether the antenna works or not has little influence on the performance of the dye-sensitized solar cells. The array antenna integrated with dye-sensitized solar cells has a similar radiation performance to the traditional microstrip slot array antenna, and can also provide electricity. Compared with the existing circularly polarized microstrip slot array antenna, the proposed antenna adds the output of dye-sensitized solar cells into the voltage regulation circuit to form a stable power supply to the radio frequency system, which ensures the operation of the microstrip slot array antenna. In other words, the antenna has the ability of self-sustaining power generation capabilities, so as to provide reliable and long-term communication for the communication system when the power is not easy to obtain.

## Figures and Tables

**Table 1:** Solar cell material properties.

Material	Dielectric Constant	Conductivity
Polyimide	3.4	3.92 × 10^−15^ S/m
Pt	1	9.3 × 10^6^ S/m
Dye-sensitized layer	90	1.51 × 10^−2^ S/m
TiO_2_	86	100 S/m
Ti	1	1.82 × 10^6^ S/m
SiO_2_	4	0 S/m
